# Comparative plastome assembly of the yellow ironweed (*Verbesina alternifolia*) using Nanopore and Illumina reads

**DOI:** 10.3389/fpls.2024.1429494

**Published:** 2024-09-12

**Authors:** Salvatore Tomasello, Eleonora Manzo, Kevin Karbstein

**Affiliations:** ^1^ Department of Systematics, Biodiversity and Evolution of Plants (with Herbarium), University of Göttingen, Göttingen, Germany; ^2^ Department of Biogeochemical Integration, Max Planck Institute for Biogeochemistry, Jena, Germany

**Keywords:** Asteraceae, chloroplast genome, Nanopore sequencing, tribe Heliantheae, *Verbesina*

## Abstract

Chloroplast genomes (plastomes) represent a very important source of valuable information for phylogenetic and biogeographic reconstructions. The use of short reads (as those produced from Illumina sequencing), along with *de novo* read assembly, has been considered the “gold standard” for plastome reconstruction. However, short reads often cannot reconstruct long repetitive regions in chloroplast genomes. Long Nanopore (ONT) reads can help bridging long repetitive regions but are by far more error-prone than those produced by Illumina sequencing. *Verbesina* is the largest genus of tribe Heliantheae (Asteraceae) and includes species of economic importance as ornamental or as invasive weeds. However, no complete chloroplast genomes have been published yet for the genus. We utilized Illumina and Nanopore sequencing data and different assembly strategies to reconstruct the plastome of *Verbesina alternifolia* and evaluated the usefulness of the Nanopore assemblies. The two plastome sequence assemblages, one obtained with the Nanopore sequencing and the other inferred with Illumina reads, were identical, except for missing bases in homonucleotide regions. The best-assembled plastome of *V. alternifolia* was 152,050 bp in length and contained 80, 29, and four unique protein-coding genes, tRNAs, and rRNAs, respectively. When used as reference for mapping Illumina reads, all plastomes performed similarly. In a phylogenetic analysis including 28 other plastomes from closely related taxa (from the *Heliantheae alliance*), the two *Verbesina* chloroplast genomes grouped together and were nested among the other members of the tribe Heliantheae s.str. Our study highlights the usefulness of the Nanopore technology for assembling rapidly and cost-effectively chloroplast genomes, especially in taxonomic groups with paucity of publicly available plastomes.

## 1 Introduction

Chloroplasts (cp) are the most emblematic organelles of plant cells, responsible for plant photosynthesis and therefore growth and reproduction. cp genomes (plastomes) are often highly conserved throughout land plants in terms of structure, size, and functionality of their genes (Bendich, 2004). Its molecule can be linear or circular (Bendich, 2004) and has a quadripartite structure consisting of two regions of unique DNA (i.e., large and small single-copy regions; LSC and SSC, respectively) and a pair of nearly identical inverted repeat regions (IRB and IRA; Kolodner and Tewari, 1979). Due to their conserved structure, low levels of recombination, and high copy numbers in plant cells, plastomes are an easily accessible source of sequence information for phylogenetic and biogeographic studies (Twyford and Ness, 2017; Tomasello et al., 2020; Karbstein et al., 2022). The advent of second-generation sequencing (e.g., Illumina sequencing) has made the assembly of entire plastomes relatively accessible, and the last decade has registered a proliferation of phylogenetic studies based on plastome data (see Tonti-Filippini et al., 2017; Pascual-Díaz et al., 2021; Dong et al., 2023). To date, more than 40,000 plant chloroplast genomes are publicly available in NCBI’s GenBank (https://www.ncbi.nlm.nih.gov/genbank/).

The genome assembly of Illumina reads has improved substantially in the past decade, and a few pipelines have been described especially dedicated to *de novo* assembly of organellar genomes (Twyford and Ness, 2017; Freudenthal et al., 2020). Fast-Plast (available at https://github.com/mrmckain/Fast-Plast/), NOVOplasty (Dierckxsens et al., 2017), and GetOrganelle (Jin et al., 2020) are some of the most widely used. These approaches usually need a certain amount of input data (>1 gigabases, Gbp; genome skimming data for GetOrganelle; https://github.com/Kinggerm/GetOrganelle) and are not always able to yield accurate assemblies when confronted with long repeat regions in chloroplast genomes (Zhou et al., 2023). In a comparison between different chloroplast genome assembly tools, GetOrganelle outperformed the others (Freudenthal et al., 2020).

Long reads, as those produced by third-generation sequencing techniques [i.e., Oxford Nanopore Technologies (ONT) and Pacific Biosciences (Pacbio)], are able to bridge long repetitive regions and therefore are helpful for plastome assembly (Pucker et al., 2022). On the other hand, these approaches are more prone to errors (Rang et al., 2018). In contrast to Pacbio, some errors in ONT sequencing seem to be non-random. Deletion errors, which are the most common errors found in Nanopore reads, increase in homonucleotide regions (Laehnemann et al., 2016). *A*-*T* miscall errors in ONT reads are less likely than all other substitution errors (Twyford and Ness, 2017; Scheunert et al., 2020).

Although several tools are available for *de novo* assembly using long-read and hybrid (both short- and long-reads) data [among others canu (Koren et al., 2017), unicycler (Wick et al., 2017), and flye (Kolmogorov et al., 2019; Syme et al., 2021)], fewer tools especially dedicated to the assembly of organellar genomes have been developed. Organelle_pba (Soorni et al., 2017) performs *de novo* assembly of any organellar (chloroplast or mitochondrial) genomes using Pacbio reads. MitoHiFi is addressing the assembly of mitochondrial genomes for a wide range of organisms (including plants) using Pacbio HiFi reads (Uliano-Silva et al., 2023). The newly described ptGAUL (Zhou et al., 2023) is dedicated specifically to plastomes, and it is able to use ONT and Pacbio reads.

*Verbesina* L. is the largest genus of tribe Heliantheae Cass. (Asteraceae), comprehending more than 325 species (Panero, 2007). It is a very diverse genus, including trees of montane moist forests, shrubs, and perennial (but also a few annual) herbs. It is distributed in Central America, the tropical Andes, and eastern Brazil, with a few taxa in the temperate regions of North and South America (Panero and Strother, 2021). Most of the species’ diversity is concentrated in Mexico and south-western USA. A few species of *Verbesina* are of big economic value as ornamentals [e.g., *V. encelioides* (Cav.) Benth. & Hook.f. ex A.Gray, *V. alternifolia* (L.) Britton ex Kearney] or are weeds with a negative impact on ecosystems in different areas of the world (Feenstra and Clements, 2008; Fufa et al., 2022; Mehal et al., 2023).

The last revision of the genus was done in the 19^th^ Century (Robinson and Greenman, 1899). A modern, comprehensive revision of the genus, including DNA-based phylogenetic evidence, is still missing (Panero and Strother, 2021). Recently, Lopes Moreira et al. (2023) analyzed a wide number of species of the genus using two multi-copy nuclear markers. A chloroplast genome for the genus is still missing, and so far, sequence information is available only for a few plastid regions of a number of *Verbesina* species (Funk et al., 2005).

With the present contribution, we aim to assemble the first chloroplast genome of a *Verbesina* species. For the scope, we utilized Illumina sequencing and long reads produced with Nanopore technology. We used different assembly strategies/tools and evaluated the correctness of the Nanopore assemblies. We evaluated the efficiency of the assembled plastomes as reference for reads mapping and reconstructed a phylogenetic tree with available plastome information from various members of the *Heliantheae alliance*.

## 2 Materials and methods

### 2.1 Plant material and DNA extraction

Leaf material from *Verbesina alternifolia* cultivated at the Old Botanical Garden of the University of Göttingen was collected in summer 2023 and silica-gel-dried. Genomic DNA was extracted from ~1.5-cm² leaf material of silica-dried samples using Qiagen DNeasy Plant Mini Kit^®^ (Qiagen, Hilden, Germany). We followed the manufacturer’s instructions, except for the incubation times in the lysis buffer and elution buffer, which were both increased to 30 min. DNA quality and fragment length were checked by gel electrophoresis in 1.5% agarose gel and using the Midori Green Advance DNA stain (NIPPON Genetics EUROPE, Düren, Germany) and the Quantitas Pro DNA Marker 100 bp–10 kb (Biozym Scientific GmbH, Hessisch Oldendorf, Germany). DNA concentration was estimated using 2 µL of extract and the Qubit^®^ fluorometer with the Qubit^®^ dsDNA HS Assay Kit (ThermoFisher Scientific, Waltham, USA).

### 2.2 Illumina library prep and sequencing

A sequencing library was prepared using the “NEBNext Ultra II FS DNA Library Prep Kit for Illumina” (E7805; New England BioLabs, Ipswich, USA). Fragmentation was carried out for 12 min at 37°C in order to obtain DNA fragments of 300–500 bp. At the end of the library preparation procedure, the sample was PCR-amplified for 14 cycles, during which sample-specific dual indices (“NEBNext Multiplex Oligos for Illumina^®^”, E7600; New England BioLabs) were added to the fragments. The library was purified with 50 μL of HighPrep PCR beads (MagBio, Gaithersburg, USA), following the manufacturer’s protocol. Concentrations were measured with the Qubit^®^ fluorometer, and fragment length distributions and absence of adapter dimers were checked using a Quiagen Qiaxcel and a high-resolution cartridge (Qiagen, Hilden, Germany).

Sequencing was conducted at the NGS- Integrative Genomics Core Unit (NIG; University of Göttingen) on an Illumina NovaSeq6000 (Illumina Inc.) SP 300 cycles flow cell. The sample was mixed equimolarly with other samples in order to gather approximately 5–7.5 Gbp of data after sequencing.

### 2.3 Nanopore library prep and sequencing

Library preparation was conducted using the ONT Ligation Sequencing Kit SQK-LSK110 optimized for high-throughput and long reads (ONT, Oxford, UK) and applicable for singleplex gDNA sequencing. We adjusted the DNA concentration to 1,000 ng in 47 µL (ca. 21.5 ng/µL). We followed the manufacturer’s instructions for library preparation (protocol vers. GDE_9141_v112_revH_01Dec2021, accessible via community.nanoporetech.com) with the few modifications applied in Karbstein et al. (2023). Accordingly, incubation times were increased up to 15 min, ethanol wash buffer concentration was increased to 80%, and we enriched the DNA fragments of more than 3 kilobases (kb).

A hardware check was performed prior to the run. We used a MinION Mk1B device and the ONT software MinKNOW vers. 21.11.9 installed on a local Linux system. We loaded the libraries into a R9.4.1 flow cell following the manufacturer’s instructions for priming and loading. Sequencing was run for 72 h.

### 2.4 Processing Nanopore data

Basecalling was done on the local HPC cluster of the University of Göttingen, (GWDG, Göttingen, Germany) using ONT software GUPPY vers. 6.0.1 and the configuration file “dna_r9.4.1_450bps_hac.cfg” (i.e., “high accuracy” basecalling). Fastq files were then appended to a single file, which was submitted to PORECHOP vers. 0.2.4 for adapter trimming (available at https://github.com/rrwick/Porechop), with the discard_middle option turned on. Trimmed reads were then subjected to length- and quality-filtering using CHOPPER vers. 0.2.0 (De Coster and Rademakers, 2023), discarding all reads shorter than 500 bp (–minlength 500). Two datasets were produced with average reads quality thread equal to or higher than 8 (-q 8) and 9 (-q 9), respectively.

Assembly was performed with CANU vers. 2.2 (Koren et al., 2017) on both datasets. For the scope, the genome size was set to 155 kilobases (kb), and the minOverlapLength was set to 500 bp. The correctedErrorRate, which is the allowed difference in overlap between two corrected reads, was set to 0.134, as suggested by the developers for high-sequencing-depth Nanopore data.

Assembled contigs were blasted against the plastome of *Helianthus annuus* L. (NC_007977.1) using BLASTN vers. 2.5.0 (Zhang et al., 2000). Contigs mapping to the *Helianthus* plastome and longer than 50 kb were aligned. Therefore, a consensus sequence was produced, and IUPAC codes were used in case of incongruences. Once this procedure was done for both the q8 and q9 datasets, the obtained sequences were aligned, and the incongruences were resolved by looking at the base called on the consensus sequence without ambiguity. In cases where the difference involved homonucleotides, the solution with the lowest number of repeated nucleotides was selected and considered the most conservative solution.

In addition to the above-mentioned procedure, we performed plastome assembly with the newly described pipeline ptGAUL (Zhou et al., 2023) (available at https://github.com/Bean061/ptgaul). ptGAUL is able to assemble plastid genomes using long-read data (similarly to GetOrganelle for Illumina reads). It is one of the very few available pipelines for this purpose, and it is able to work both with Nanopore and PacBio data. We used the plastome of *H. annuus* (NC_007977.1) as reference and the default settings, apart from the coverage (-c), which was set to 500.

### 2.5 Processing Illumina reads and annotation

Illumina reads were *de novo*-assembled using the software GetOrganelle vers. 1.7.7.0 (Jin et al., 2020), specifying the “embplant_pt” database and with the k-mer size ranging from 21 to 115. The maximum number of extension rounds was set to 30, and the maximum number of reads (–max-reads) was increased to 7.5e7. The average embplant_pt base coverage was 1,144.

The plastome from GetOrganelle was annotated using the online tool GeSeq (Tillich et al., 2017) (available at https://chlorobox.mpimp-golm.mpg.de/geseq.html). The BLAT (Kent, 2002) searches were done by setting the protein search identity to 80% and the rRNA, tRNA, and DNA search identity to 85%. As reference plastome, we used the *H. annuus* plastome also used in the above-mentioned analyses (NC_007977.1). Additionally, the MPI-MP land plant references were used for chloroplast CDS and rRNAs. For tRNA annotation, a HMMER profile search was also done using ARAGORN vers. 1.2.38 (Laslett and Canback, 2004) with the default settings. The obtained annotation was checked manually in Geneious Prime 2022.1.1 (https://www.geneious.com). The final annotated chloroplast genome was converted into a graphical maps using OGDRAW (Greiner et al., 2019; available at https://chlorobox.mpimp-golm.mpg.de/OGDraw.html) with default settings.

### 2.6 Read mapping using the assembled plastomes as reference

To evaluate the performance of the plastomes obtained with the different sequencing technologies when acting as reference, we mapped the Illumina reads against the two plastome sequences (i.e., obtained from Illumina and Nanopore sequencing) using BOWTIE2 vers. 2.3.5.1 (Langmead and Salzberg, 2013) and BWA vers. 0.7.16 (Li and Durbin, 2009). Before mapping, the fastq files were processed to trim adaptors and filter low-quality reads with TRIMMOMATIC vers. 0.33 (Bolger et al., 2014). Duplicate reads were also excluded using FASTUNIQ (Xu et al., 2012). After mapping, we compared the percentage of reads mapping to the reference and the average coverage.

### 2.7 Phylogenetic analyses

In order to evaluate the correct phylogenetic position of the two reconstructed plastomes (i.e., from Illumina and Nanopore sequencing) in relation to those of closely related organisms and check the possible effects of the differences between them, a phylogenetic analysis was conducted. A total of 28 chloroplast genomes of taxa closely related to *Verbesina alternifolia* (from the *Heliantheae alliance*) were downloaded from GenBank and aligned with the two obtained plastomes (i.e., from Illumina and Nanopore sequencing). A complete list of the accessions used is given in **Table 1**. In some cases, the orientation of the small single copy region (SSC) was inverted in order to make it match to the other accessions (see **Table 1**). *Senecio vulgaris* L. (NC_046693.1) was included and used as outgroup. The sequences were processed in AliView vers. 1.20 (Larsson, 2014) and aligned with MAFFT vers. 7.305b (Katoh and Standley, 2013), with the –auto strategy. The final alignment consisted of 163,123 characters, and the region between positions 140,777 and 141,391 was masked due to a long insertion in the plastome of *Parthenium argentatum* A.Grey (NC_013553.1) that made an unambiguous alignment of the rest of the accessions impossible. A maximum likelihood (ML) phylogenetic tree was inferred with RAXML-NG vers. 1.2.0 (Kozlov et al., 2019), using the GTR+G as sequence evolution model and applying 1,000 bootstrap replicates.

**Table 1:** Detailed information on the plastomes used for the phylogenetic analysis in **Figure 2**.

Taxon	GenBank accession no.	Subtribe	Tribe
*Acmella paniculate*	MZ_292978	Spilanthinae	Heliantheae
*Aldama grandiflora*	MN_337894	Helianthinae	Heliantheae
*Ambrosia trifida*	NC_036810	Ambrosiinae	Heliantheae
*Bidens parviflora*	MW_691204	Coreopsidinae	Coreopsideae
*Dahlia pinnata*	NC_066129*	Coreopsidinae	Coreopsideae
*Echinacea pallida*	NC_034321	Zinniinae	Heliantheae
*Echinacea purpurea*	NC_034327	Zinniinae	Heliantheae
*Eclipta alba*	NC_039774*	Ecliptinae	Heliantheae
*Eclipta prostate*	NC_030773*	Ecliptinae	Heliantheae
*Gaillardia pulchella*	OR_124734*	Gaillardiinae	Helenieae
*Galinsoga parviflora*	NC_046787	Galinsoginae	Millerieae
*Helianthus annuus*	NC_007977*	Helianthinae	Heliantheae
*Helianthus tuberosus*	MG_696658	Helianthinae	Heliantheae
*Iostephane heterophylla*	MT_700542	Helianthinae	Heliantheae
*Pappobolus lanatus*	MT_700543	Helianthinae	Heliantheae
*Parthenium argentatum*	NC_013553	Ambrosiinae	Heliantheae
*Parthenium hysterophorus*	MT_576959	Ambrosiinae	Heliantheae
*Rudbeckia hirta*	OR_124735	Rudbeckiinae	Heliantheae
*Rudbeckia laciniata* var. *laciniata*	MN_518844	Rudbeckiinae	Heliantheae
*Sigesbeckia orientalis*	MN_240004	Milleriinae	Millerieae
*Silphium integrifolium*	OM_162161	Engelmanniinae	Heliantheae
*Silphium perfoliatum*	NC_060408*	Engelmanniinae	Heliantheae
*Smallanthus sonchifolius*	NC_072537	Milleriinae	Millerieae
*Sphagneticola calenduladea*	NC_039346*	Ecliptinae	Heliantheae
*Tithonia diversifolia*	MT_700544	Helianthinae	Heliantheae
*Xanthium sibiricum*	MH_473582	Ambrosiinae	Heliantheae
*Xanthium spinosum*	NC_054222	Ambrosiinae	Heliantheae
*Senecio vulgaris*	NC_046693		Senecioneae

The plastome of Senecio vulgare, not a member of the Heliantheae alliance, was used as outgroup. The asterisk symbols are for sequences in which the SSC was inverted in the alignment used for inferring the phylogenetic tree shown in **Figure 2**.

## 3 Results

### 3.1 Nanopore sequencing

Sequencing produced 116.81 Gigabytes (Gb) of *fast5 files (986 files in total). Furthermore, 3.94 million reads were generated with a median read fragment length of 7.11 kb. The longest read was 630 kb, and the overall data produced amounted to 11.14 Gbp. After basecalling, 1,875,797 reads were present in the fastq file, and 1,875,688 reads survived adapter trimming. After length- and quality-filtering, 1,349,347 and 1,349,205 reads were still present when using –q 8 and –q 9 as quality thresholds, respectively (corresponding to approximately 5.473 Gbp of sequence data).

The plastome assembled using our custom procedure (i.e., assembly with canu, blastn, concatenation of plastome contigs; see “Materials and methods” section) was 151,795 bp, with 45 unresolved ambiguities, including 43 N, one *M* (*A* or *C*), and one *Y* (*T* or *C*). ptGAUL produced two results (paths), corresponding to the different states caused by the differing orientations of the small single copy (SSC) region [“flip-flop” (Palmer, 1983; Walker et al., 2015)]. The plastome assembled with ptGAUL was 151,829 bp in size (see **Table 2**).

**Table 2:** Summary of the assemblies obtained with reads from different sequencing technologies and with different approaches (for the Nanopore reads).

	Illumina	Nanopore	
	GetOrganelle	Custom procedure	ptGAUL
Total length	152,050	151,795	151,829
A	47,205	47,103	47,129
C	28,264	28,218	28,235
T	47,542	47,434	47,463
G	29,039	28,995	29,002
N	0	43	0
y (instead of t)		1 (at position 13,091)*	
m (instead of c)		1 (at position 110,091)*	
Total changes		300	221
% dissimilarity		0.197	0.145

### 3.2 Illumina sequencing

Approximately 25.7 million pairs of reads were generated by Illumina sequencing, corresponding to 7.7 Gbp of data. GetOrganelle obtained a circular plastid genome of 152,050 bp in two different states. This plastome is available on GeneBank with accession number PP639077.

When comparing the plastome obtained from Illumina reads to those from the Nanopore sequencing, the GetOrganelle plastome was 221 and 255 bp longer than the ones obtained with ptGAUL and with our custom procedure, respectively. The fewer basepairs found in the Nanopore plastomes corresponded to the bases missing in homonucleotides regions. The same is true for the *N* in the plastome reconstructed with the custom procedure, which were all found in homonucleotide regions. The *M* in the latter plastome corresponded to a *C* in the other two plastomes, whereas the *Y* corresponded to a *T* (**Table 2**). In approximately 60% of the cases, bases missing from homonucleotide regions were found in the same regions of the two plastomes inferred with ONT data. In approximately 50% of cases, the number of missing bases was identical (including gaps as long as seven basepairs). The alignment with the three plastomes inferred (i.e., one from Illumina data and two from Nanopore reads) is available on Göttingen Research Online (GRO.data; doi:10.25625/QJCSC8).

### 3.3 Annotation

**Figure 1:**
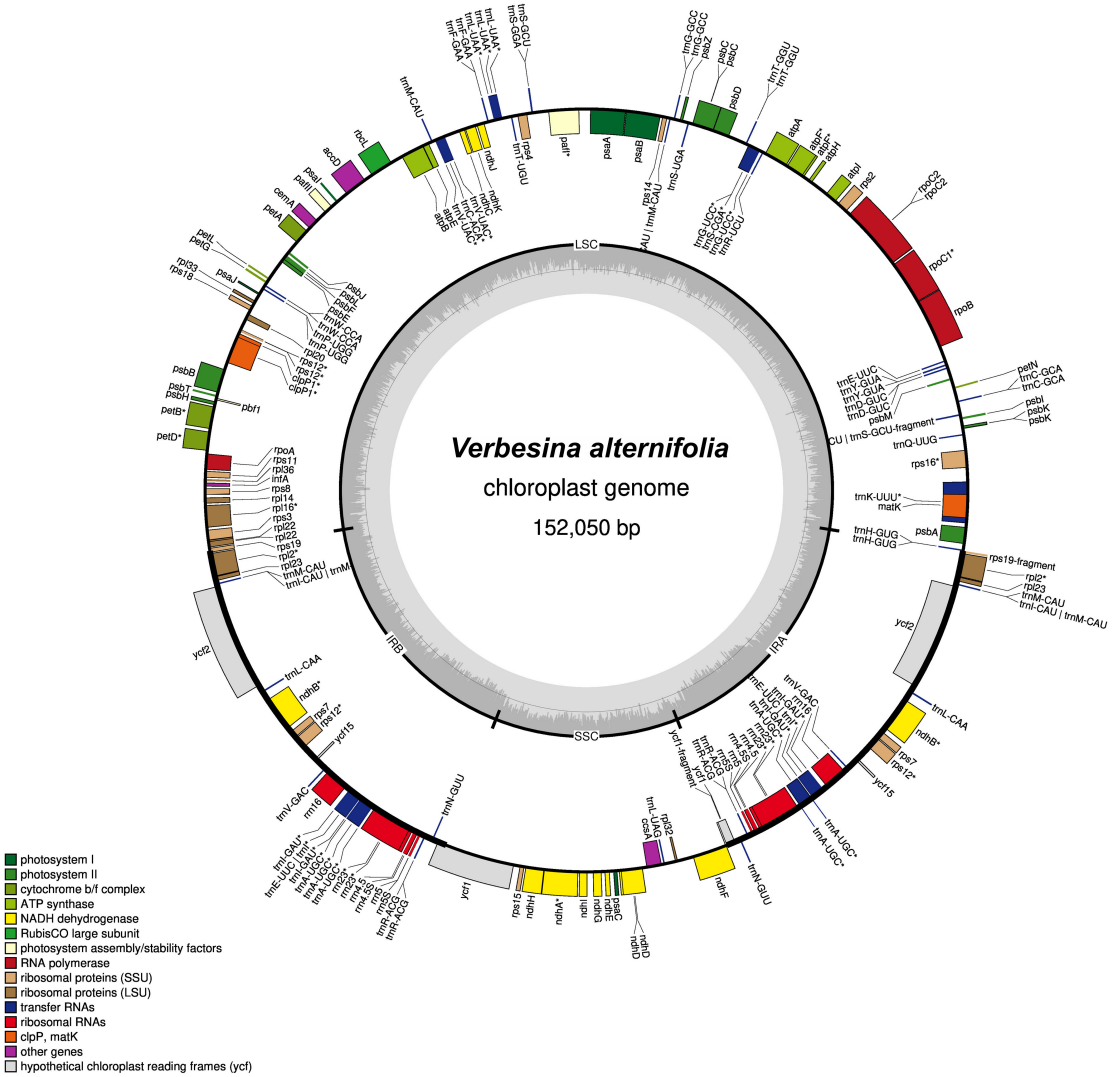
Chloroplast genome map for *Verbesina alternifolia*. The color of genes indicates their affiliation to the functional groups. The first inner circle depicts the genome structure with the small single-copy region (SSC), the large single-copy region (LSC), and the two inverted repeats (IRA/B). The innermost gray-shaded circle represents the GC content (%). Genes on the inside of the map are transcribed in the clockwise, whereas genes on the outside counterclockwise. Genes containing an intron are marked with an asterisk (*).

The annotated plastome shows a typical tripartite structure with a small single-copy region (SSC, length: 18,241 bp) and a large single-copy region (LSC, length: 83,721 bp) separated by two IR regions (length: 2 × 25,044 bp; **Figure 1**). The plastome contains 86 (80 unique) protein-coding genes, 36 (29 unique) tRNAs, and eight (four unique) rRNAs (duplicated ones in IRs).

In comparison with the *Helianthus annuus* plastome (NC_007977.1), the *Verbesina alternifolia* chloroplast genome (the one here reconstructed with GetOrganelle) was 969 bp longer. It has the same numbers of unique protein-coding genes and rRNA (80 and four, respectively) and slightly more unique tRNAs (29 vs. 28).

### 3.4 Mapping performance

Mapping of Illumina reads produced comparable results when using the different references (i.e., the plastomes obtained from Illumina and ONT reads) and mapping methods (**Table 3**). BWA seems to have performed slightly better than BOWTIE2 in terms of mapped reads (560,311.5 and 537,273.5 reads mapped on average, respectively). When comparing the performance as reference, the plastome obtained with the Illumina reads gave slightly higher numbers of mapped reads (565,237 vs. 546,310 reads), although average per-base coverage was better when using the Nanopore reference (134.055 vs. 128.996). Detailed results are given in **Table 3**.

**Table 3:** Comparison of the mapping results for the short Illumina reads when using as reference the plastomes assembled with Illumina or Nanopore reads and two different mapping programs.

Reference plastome (mapping tool)	Nr. reads	Paired reads	Forward unpaired	Reverse unpaired	Mapped reads	% mapped	mean coverage
Illumina (bowtie2)	43,120,986	21,086,222	782,412	166,130	537,317	1.246	128.360
Illumina (bwa)					565,237	1.31	129.632
ONT custom procedure (bowtie2)					537,234	1.245	128.555
ONT custom procedure (bwa)					555,386	1.287	139.555

“Nr. reads” refers to the number of reads after quality trimming and duplicate reads removal.

### 3.5 Phylogenetic analyses

**Figure 2:**
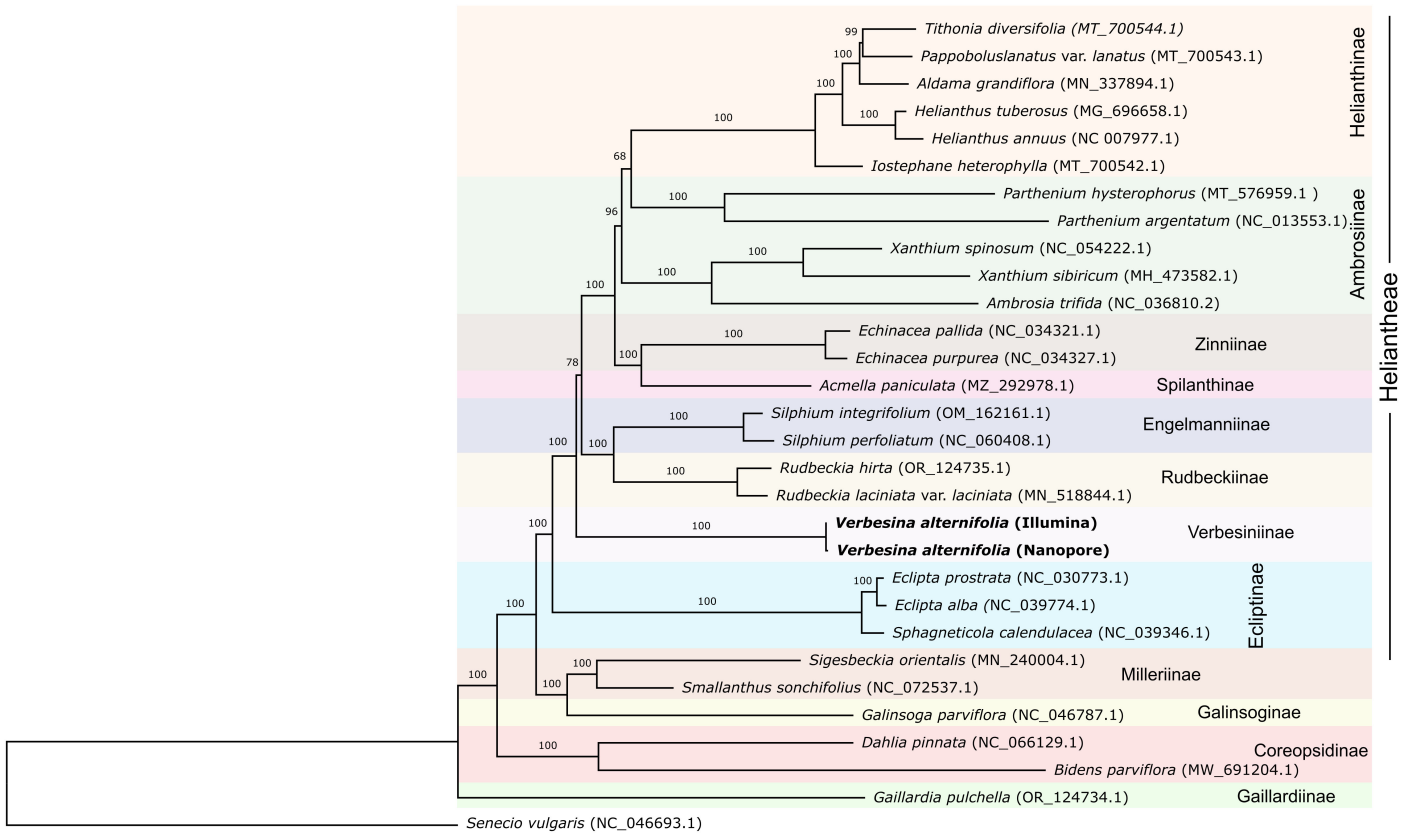
ML phylogenetic tree including *Verbesina* plastomes assembled from Illumina and Nanopore reads, along with plastomes from members of the *Heliantheae alliance* available on the GenBank (**Table 1**). The numbers above the branches are bootstrap support values. On the right part of the figure, the subtribal membership of the accessions is indicated. Colors are according to subtribes in the *Heliantheae alliance*.

The phylogenetic tree obtained from the ML analyses was fully resolved, with just a couple of clades receiving relatively low bootstrap (bs) support values (bs <90; **Figure 2**). The two *Verbesina* plastomes (i.e., Illumina and ONT assemblies) grouped together with high support (bs: 100). Those are found within tribe Heliantheae, sister to a clade including subtribes Rudbeckiinae H.Rob., Engelmanniinae Stuessy, Spilathinae Panero, Zinniinae Benth. & Hook.f., Ambrosiinae Less., and Helianthinae Dumort (clade, however, moderately low supported; bs: 78).

Additionally, the clade including Helianthinae and *Parthenium* L. (from subtribe Ambrosiinae) is not supported (bs: 68). Subtribe Ambrosiinae is not reconstructed as monophyletic, with genera of the “core” Ambrosiinae (here *Xanthium* L. and *Ambrosia* L.) grouping together with high support and *Parthenium* sister to subtribe Helianthinae (relationship, however, not supported).

## 4 Discussion

Insights gained from chloroplast genomes have enhanced our understanding of plant biology and diversity (Daniell et al., 2016). On the one hand, in the last few decades, the use of a few variable plastid regions has served to resolve phylogenies at a relatively deep taxonomic level (Wicke and Quandt, 2009; Dong et al., 2015). On the other, complete chloroplast genomes provide the high resolution necessary to differentiate closely related taxa and are therefore a valuable source of information to decipher phylogenetic relationships between them and to improve our understanding of the evolution of plant species (Daniell et al., 2016).

Third-generation sequencing, with the long reads it can produce, facilitates *de novo* genome assembly of plastid genomes, particularly in the four junctions between the inverted repeat (IR) and single-copy regions (Daniell et al., 2016). Long reads can also help in assembling plastid genomes in plant groups characterized by rampant plastome rearrangements (see Wicke et al., 2011 for a review). Nanopore technology has demonstrated to be a good solution for sequencing and assembling plastid genomes in plant groups for which this information is still missing (Bae and Kim, 2021; Scheunert et al., 2020; among others).

With the present contribution, we assembled the first chloroplast genome for the genus *Verbesina*. *Verbesina* is the most species-rich genus of tribe Heliantheae (Asteraceae), with a number of species important as ornamental or because of their invasive attitude. For the scope, we have used both short Illumina reads and long Nanopore ones. We have assembled chloroplast genomes using different approaches and evaluated the effectiveness of the Nanopore reads against the Illumina data. Our study highlights the importance of Nanopore technology as a tool to rapidly and cost-effectively assemble plastid genomes, particularly in taxonomic groups in which no plastomes are publicly available yet.

### 4.1 Illumina vs. Nanopore assemblies

The plastome assembled with Nanopore data resulted very similar to the one inferred with Illumina reads by GetOrganelle. In the plastome assembled with our custom procedure (see “Materials and methods”), only two positions were not unambiguously resolved, whereas the plastome reconstructed with ptGAUL was identical to the Illumina genome, apart from missing bases in homonucleotide regions. When considering gaps (missing bases in homonucleotide regions), the sequence identity of the former and latter plastomes to the one obtained with Illumina reads is 99.80% and 99.85%, respectively (see **Table 2**). These values are in line with those obtained in comparable studies [e.g., 99.59% in Scheunert et al. (2020)].

All mismatches (all but two in the Nanopore plastome assembled with our custom procedure) were gaps in homonucleotide regions. In approximately 60% of cases, these gaps were found in the same homonucleotide regions in both Nanopore plastomes; in approximately 50% of cases, these gaps were identical in terms of missing nucleotide, including extreme cases of gaps with up to seven missing nucleotides (see alignment available at https://doi.org/10.25625/QJCSC8). If, on the one hand, this confirms what has already been found in other studies, that Nanopore sequencing accumulates deletion errors in homonucleotide regions (Laehnemann et al., 2016; Scheunert et al., 2020), it also testifies that this accumulation does not depend on the assembly strategy used. It must be also noted that since couple of years the R10.4.1 flow cell has been released, which has contributed to improving the read quality and the results of assemblies, although homopolymer problems persist to some extent (Lerminiaux et al., 2024; Sawicki et al., 2024). However, such mismatches are unimportant in phylogenetic analyses since most of the phylogenetic methods do not take gaps into account (Husmeier, 2005; Mahadani et al., 2022). This is also reflected in our phylogenetic analyses, in which (as expected) the *Verbesina* plastomes assembled from reads obtained with different sequencing technologies clustered together and with full support in the phylogenetic tree (**Figure 2**).

When comparing the two approaches used for the assembly of Nanopore reads, ptGAUL clearly outperformed our custom approach (i.e., assembly with canu, blastn, and concatenation of plastome contigs). ptGAUL managed to assemble a circular molecule, whereas canu was unable to recover the plastome in a single contig. This is something already noticed in other studies (Wang et al., 2018; Scheunert et al., 2020) in which the *de novo* assembly of plastomes often resulted in two or more contigs of varying length. There might be multiple reasons for that: the high coverage of our Nanopore sequencing may result in alternative assemblies which can lead to contig fragmentation (Izan et al., 2017; Wang et al., 2018); the quality the quality of the DNA (Bethune et al., 2019), i.e., we used silica-gel-dried leaf material and an extraction protocol not specifically designed for the extraction of ultra-long fragments.

A chloroplast genome inferred with Nanopore data might also be important as reference in taxonomic groups, in which no other plastomes are publicly available yet. For instance, *de novo* assemblers of plastomes based on Illumina data need a certain sequencing depth (>1 Gbp of genome skimming data as suggested by the developers of GetOrganelle). The amount of data needed for *de novo* assembly may vary depending on different factors, e.g., factors intrinsic to the taxonomic group under investigation (e.g., nuclear genome size), the type of material (degraded DNA and archival DNA), or technical issues (library preparation protocol). In such circumstances, even more than 1 Gbp of genome skimming data might not be enough to reconstruct complete plastid genomes using short reads data. A reference plastome obtained with long Nanopore reads may serve for reference-based mapping of Illumina short reads with, e.g., BWA or BOWTIE2, when the amount of Illumina data available is not enough to reconstruct complete plastomes with the above-mentioned *de novo* approaches.

### 4.2 Phylogenetic considerations

Inference of phylogenetic relationships among higher-level classification lineages and the identification of clear borders of tribe Heliantheae have been challenging due to extensive variations in morphological characters registered in members of this taxonomic group and the complex patterns that sometimes result from molecular-based phylogenetic analyses (Panero, 2007; Baldwin, 2009). Heliantheae is now treated as a group (alliance), including a few major lineages recognized at tribal rank (Panero and Funk, 2002), Heliantheae s.str. being one of them. The relationships among the tribes that we included in this study (**Figure 2**) reflect the one depicted in Panero (2007) (also based on cp data) and in Zhang et al. (2021) (based on transcriptomic data), with Helenieae Lindl. (here represented by *Gaillardia* Foug.) as an early branch in the alliance and then Coreopsideae Lindl. (*Bidens* L. and *Dahlia* Cav.) and Millerieae Lindl. (*Galinsoga* Ruiz & Pav., *Smallanthus* Mack., *Sigesbeckia* L.) as sister to the Heliantheae s.str. Within tribe Heliantheae, the position of subtribes is again congruent with the cpDNA tree shown in Panero (2007), with Verbeseninae being sister to a relatively big clade including Rudbeckiinae, Engelmanniinae, Spilanthinae, Zinniinae, Ambrosiinae, and Helianthinae (**Figure 2**). The supposed sister relationship between Verbesiniinae and Engelmanniinae based on transcriptomic data (Zhang et al., 2021) is not supported here.

Interestingly, subtribe Ambrosiinae resulted paraphyletic, with *Parthenium* nested rather in the clade with members of subtribe Helianthinae (**Figure 2**). The non-monophyly of subtribe Ambrosiinae was already supposed (Panero, 2005) and supported by cp DNA data (Panero et al., 2001; Panero, 2007; Tomasello et al., 2019). Nuclear DNA-based analyses, however, seem to sustain the monophyly of the subtribe (Tomasello et al., 2019; Zhang et al., 2021).

## Data Availability

The datasets presented in this study can be found in online repositories. The names of the repository/repositories and accession number(s) can be found in the article/supplementary material.
